# Transcriptomic Analyses Suggest the Adaptation of Bumblebees to High Altitudes

**DOI:** 10.3390/insects13121173

**Published:** 2022-12-17

**Authors:** Chengbo Liang, Daoxin Liu, Pengfei Song, Yuantao Zhou, Hongyan Yu, Guo Sun, Xiaoxuan Ma, Jingyan Yan

**Affiliations:** 1 State Key Laboratory of Plateau Ecology and Agriculture, Qinghai University, Xining 810016, China; 2 College of Agriculture and Animal Husbandry, Qinghai University, Xining 810016, China; 3 Kunlun College, Qinghai University, Xining 810016, China; 4 Key Laboratory of Adaptation and Evolution of Plateau Biota, Northwest Institute of Plateau Biology, Chinese Academy of Sciences, Xining 810001, China; 5 Qinghai Service Guarantee Center of Qilian Mountain National Park, Xining 810001, China

**Keywords:** bumblebees, high-altitude adaptation, hypoxia, low air density

## Abstract

**Simple Summary:**

Determining the adaptive mechanisms by which bumblebees adapt to high altitudes can help us to better understand their distribution, providing a basis for the future protection and utilization of bumblebee resources. For this study, the adaptive mechanisms of two dominant bumblebee species in the northeastern Qinghai-Tibet Plateau—*Bombus kashmirensis* and *B. waltoni*—were studied through transcriptomics methods. For each species, enrichment analysis of the differentially expressed genes and gene set enrichment analysis were carried out between samples collected at different altitudes (4000 m, 4500 m, and 5000 m). The results indicate that these bumblebees tend to up-regulate energy metabolism-related genes when facing extremely high-altitude environments. Of the enriched pathways up-regulated in higher altitudes, the pentose and glucuronate interconversions pathway presented the most severe up-regulation in multiple comparisons of different altitudes for *B. kashmirensis*, as well as the AMPK signaling pathway, which was found to be up-regulated in both species. Notably, limited by the extreme high altitudes in this study, oxidative phosphorylation was found to be down-regulated with increasing altitude, which is uncommon in studies on bumblebee adaptation to high altitudes.

**Abstract:**

Determining the adaptive mechanisms by which bumblebees adapt to high altitudes can help us to better understand their distribution, providing a basis for the future protection and utilization of bumblebee resources. For this study, the adaptive mechanisms of two dominant bumblebee species in the northeastern Qinghai-Tibet Plateau—*Bombus kashmirensis* and *B. waltoni*—were studied through transcriptomics methods. For each species, enrichment analysis of the differentially expressed genes and gene set enrichment analysis were carried out between samples collected at different altitudes (4000 m, 4500 m, and 5000 m). The results indicate that these bumblebees tend to up-regulate energy metabolism-related genes when facing extremely high-altitude environments. Of the enriched pathways up-regulated in higher altitudes, the pentose and glucuronate interconversions pathway presented the most severe up-regulation in multiple comparisons of different altitudes for *B. kashmirensis*, as well as the AMPK signaling pathway, which was found to be up-regulated in both species. Notably, limited by the extreme hypoxic conditions in this study, oxidative phosphorylation was found to be down-regulated with increasing altitude, which is uncommon in studies on bumblebee adaptation to high altitudes.

## 1. Introduction

As one of the most important wild pollinators in both cultivated and natural ecosystems in the northern temperate world, bumblebees (*Bombus* spp.) play a critical role in maintaining the balance of various ecosystems [1,2,3,4]. Although bumblebees are distributed in a wide range of diverse habitats, some of them are most abundant in alpine and high-elevation areas, showing strong adaptation to high altitudes [5]. In alpine zones, due to the limited activities of butterflies, beetles, and other pollinators under extreme conditions, bumblebees have become key irreplaceable pollinators [2,3]. Studies on bumblebee biodiversity in many alpine regions have been conducted by different researchers, for the purpose of effective high-altitude bumblebee resource management and conservation [6]. Knowledge on the mechanisms through which bumblebees adapt to high altitudes can help us reach a better understanding regarding their current distribution and predict future range shifts driven by changing climate, as well as providing meaningful information for the protection of bumblebee resources.

The high-altitude adaptation of animals has long been a hot topic, widely investigated by researchers. To face the challenges under high-altitude environments, mammals at high altitudes have developed the ability to adapt to hypoxia in different ways after long-term evolution [7,8,9,10,11]. The hypoxia response pathways of humans allow for the increased delivery and reduced consumption of oxygen, in order to regulate organic energy balance by providing enough oxygen to the aerobic metabolism [12,13]. Some common characteristics, in terms of the metabolism of oxygen and production of energy, as in humans, have been demonstrated in studies on the high-altitude adaptation of dogs, pikas, and other animals [14,15,16,17,18,19]. Some of these characteristics have also been observed in certain insects, such as *Drosophila* [15]. Of course, the mechanisms for high-altitude adaptation can also differ among different organisms [18,19,20], for example, Tibetan locusts tend to exhibit a dramatic and heritable reduction in body size, compared to lowland locusts, while certain bumblebees respond to altitude in the opposite way [21].

The adaptation of bumblebees to high altitudes has already been studied and interpreted in different aspects [22]. Flight at high altitudes can be seen as a direct challenge for bumblebees. Dillon et al. [22] have paid attention to the flight of *B. impetuosus* in a flight chamber simulating the low air pressure at high altitudes, and concluded that bumblebees can overcome air with low density through increasing the stroke amplitude, and not wingbeat frequency. According to Sun et al. [23], in some high-altitude bumblebee species, positive selection was detected in specific genes, involved in eye development, maintenance of muscle integrity, keeping muscle on flight state, and metabolic adaptation to hypoxia. It has been speculated that the positive selection of genes involved in eye development may be related to facilitation of the choice of an optimal foraging path in high-altitude light conditions, which can save much flight in foraging, as bumblebees rely heavily on visual signals to detect flowers [24]; meanwhile, the other three biological processes concerning the genes under positive selection are all beneficial to flight energy supply. Liu et al. [25] have reported the up-regulated expression of genes related to aerobic and anaerobic glycolysis processes in high-altitude bumblebee species, when compared to low-altitude species. Taking *B. pyrosoma* as the experimental object, the authors further studied [26] intraspecies transcriptomic differences in high-altitude adaptation by comparing cospecies from the Qinghai-Tibet Plateau (QTP) and the North China Plain with an average altitude difference over 2500 m. In their study, not only were the metabolism and transport of energy resources enhanced, but immune defense and the Toll and immune deficiency pathways, were also enhanced at high altitudes.

According to previous studies, high-altitude adaptation is a complicated biological problem concerning many aspects, including morphology, physiology, genetics, transcriptomics, and so on. Revealing the regulation of gene expression by the transcriptome has always been considered to be one of the most effective methods for adaptation studies. Transcriptome differences in bumblebees at different altitudes demonstrate adaptation to those altitudes. Therefore, the transcriptome was chosen to study the high-altitude adaptations of bumblebees here. In this study, two widespread alpine species in the QTP [27]—*Bombus kashmirensis* and *Bombus waltoni*—were sampled at different sites with an altitude gradient of about 500 m in the respective species. The distances between collection sites were less than 100 km, in order to minimize genetic background differences. Transcriptomic differences between samples from different altitudes were analyzed in order to explore the adaptive mechanisms of these bumblebees to high altitudes. The results indicated that these bumblebees tended to up-regulate partial pathways of the energy metabolism but that the oxidative phosphorylation may unavoidably go down in extremely high-altitude environments.

## 2. Materials and Methods

### 2.1. Transcriptome of Samples

**Figure 1:**
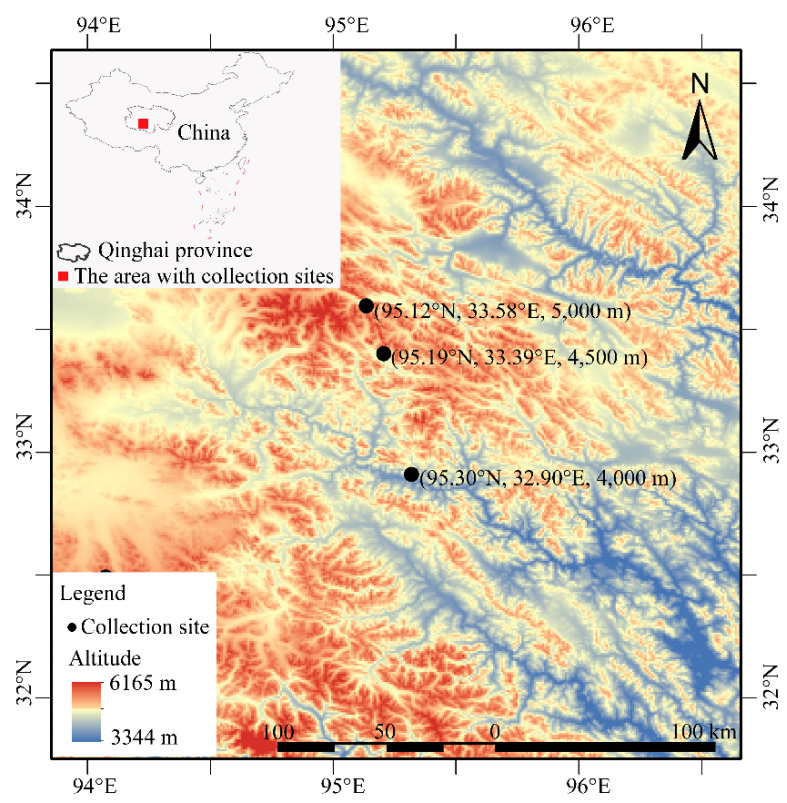
The collection sites of the two studied bumblebee species.

Sampling sites were chosen in the northeastern QTP, as this region is next to the global biodiversity center of bumblebees and a large number of species are distributed. On the other hand, the study area is close to the plateau edge and rich in high mountains, with high average altitude and severe altitude changes over a small range, making it a good choice for the study of high-altitude adaptation. Based on a previous investigation in the QTP, two indicator species, *B. kashmirensis* and *B. waltoni* [27], were chosen as the objects of this study. In order to minimize the potential influence of genetic difference caused by great altitude changes or large geographical distances, both species were collected at three collection sites along an elevation gradient including 4000 m, 4500 m, and 5000 m (Figure 1). The distance between sites was less than 100 km. The total altitude range of *B. waltoni* is about 2500~5200 m [5]. The lower limit of altitude range of *B. kashmirensis* is about 2100 m [28], and its upper limit of altitude range is not higher than 5400 m [29]. In this study, the upper part of both species’ total altitude range was covered to study the two species’ adaptation to extremely high-altitude environments. In total, seventeen workers of *B. kashmirensis*, with five, five, and seven at 4000 m, 4500 m, and 5000 m, respectively, and thirteen workers of *B. waltoni*, with five, three, and five at 4000 m, 4500 m, and 5000 m, respectively, were sampled. All of the samples were active foragers with a similar body size and were sampled from flowers on sunny days. The total RNA of each sample was extracted from the body without the abdomen, and then checked by electrophoresis in agarose gel, using a Nanodrop 2000 and Agilent 2100 after removing the abdomen of species. The qualified RNA (with mass ≥ 1 μg and concentration ≥ 35 μg/μL) was used to extract mRNA using Oligo (dT) magnetic beads (Invitrogen, Carlsbad, CA, USA). The extracted mRNA was used for synthesis of cDNA by reverse transcription and random hexamers, after treatment with fragmentation buffer (Illumina, CA, USA). Then, the ends of the cDNA were repaired using End Repair Mix (Illumina, CA, USA). During this process, a single A base and adapters were added to each end of the cDNA. Then, cDNA sequencing was carried out on an Illumina NovaSeq 6000 platform after PCR amplification. Raw data was gained from the Illumina platform by CASAVA, and has been deposited into the NCBI SRA database (*Bombus kashmirensis*: from SRR19913284 to SRR19913300; *Bombus waltoni*: from SRR19913147 to SRR19913159). Clean data were obtained from raw data by removing the adapter sequence and low-quality reads. Transcriptome assembly was accomplished by Trinity v2.8.5 (k-mer = 31) [30]. Genes were annotated based on the National Center for Biotechnology Information non-redundant protein (NCBI_NR), Swiss-Prot, Protein family (Pfam), clusters of orthologous groups of proteins (COG), Gene ontology (GO), and Kyoto Encyclopedia of Genes and Genomes (KEGG) databases using TransDecoder v5.5.0. The gene expression levels were evaluated by RSEM v1.3.1 [31], based on the transcript per million (TPM). In the following analysis, transcriptome comparisons of the two bumblebee species between different altitudes were conducted separately, and the analyses were performed using the online Majorbio Cloud Platform (www.majorbio.com (accessed on 10 March 2022)).

### 2.2. Identification of Adaptation-Related GO Terms

According to the annotation results, genes involved in GO terms were used to perform the gene set enrichment analysis (GSEA) using the Majorbio Cloud Platform (https://cloud.majorbio.com/page/tools/ (accessed on 10 March 2022)). For each species, the transcriptomic differences of 4500 m vs. 4000 m, 5000 m vs. 4500 m, and 5000 m vs. 4000 m were explored through GSEA. In each comparison, the samples from the lower altitude were set as the control group and enriched GO terms were identified at the higher elevation. The enriched GO terms with the top 20 largest absolute normalized enrichment scores (NES) and *p*-values (adjusted by the Benjamini–Hochberg approach) lower than 0.05 were focused in the following analysis.

### 2.3. KEGG Enrichment of Differentially Expressed Genes

For each species, the KEGG enrichment of differentially expressed genes (DEGs) for 4500 m vs. 4000 m, 5000 m vs. 4500 m, and 5000 m vs. 4000 m was assessed, wherein we identified up- and down-regulated genes at the higher elevation. Differentially expressed genes (DEGs) in each analysis were screened using DESeq 2 v1.24.0 [32]. The genes with a fold change more than two and *p*-value (adjusted by the Benjamini–Hochberg approach) less than 0.05 were considered as DEGs. The KEGG enrichment of differentially up- or down-regulated genes was assessed using a script developed by Majorbio (https://www.majorbiogroup.com (accessed on 10 March 2022)). The enriched KEGG pathways with *p*-values (corrected by the Benjamini–Hochberg approach) lower than 0.05 were considered as key pathways related to altitude adaptation.

## 3. Results

### 3.1. RNA-Seq and Transcriptomic Annotation

Total RNAs of all samples of *B. kashmirensis* and *B. waltoni* were qualified. Among these RNA, one of them presented an OD260/280 value of 1.98, and the others had OD260/280 values greater than two. The Q30 values of clean reads of all samples were above 90%, and the error rates were about 0.03%. The average GC content was 42.71% for *B. kashmirensis* samples and 46.32% for *B. waltoni*. The numbers of clean bases of *B. kashmirensis* and *B. waltoni* were 6.54 G and 6.74 G, respectively. The GC contents of unigenes for these two species were 40.55% and 41.67%, respectively. The mapping ratios of each *B. kashmirensis* sample computed by hisat2 v2.1.0 ranged from 69.74% to 83.32%, while that for *B. waltoni* ranged from 74.02% to 82.28% (Table 1).

### 3.2. The Gene Set Enrichment Analysis of Gene Expression

In the GSEA of *B. kashmirensis*, there were 29 enriched GO terms with adjusted *p*-values lower than 0.05 in samples from 4500 m compared to 4000 m. The terms with the top 20 largest absolute NES (normalized enrichment scores) were mainly enriched by down-regulated genes, except for protein lipidation and steroid dehydrogenase activity terms, which were mainly enriched by up-regulated genes. The protein lipidation term has a positive effect on effective protein processing. In samples from 5000 m, 120 and 50 enriched GO terms with adjusted *p*-values lower than 0.05 were discovered compared to samples from 4500 m and 4000 m, respectively. In the comparison of 5000 m vs. 4500 m, all terms with the top 20 largest absolute NES were mainly enriched by up-regulated genes while for 5000 m vs. 4000 m, only six of the terms with the top 20 largest absolute NES were mainly enriched by up-regulated genes (Table 2).

In the GSEA of *B. waltoni*, there were 45 enriched GO terms with adjusted *p*-values lower than 0.05 in samples from 4500 m compared to 4000 m. All of the terms with the top 20 largest absolute NES were mainly enriched by down-regulated genes. In the analysis of 5000 m vs. 4500 m, no GO terms presented an adjusted *p*-value lower than 0.05. In the analysis of 5000 m vs. 4000 m, based on the adjusted *p*-values, 11 enriched GO terms were significant in samples from 5000 m compared to 4000 m. Among these enriched GO terms, only the demethylation term was mainly enriched by up-regulated genes (Table 3).

In the comparison of 4500 m vs. 4000 m, the terms of chitin binding, motile cilium and odorant binding were mainly enriched by down-regulated genes in both species. In the comparison of 5000 m vs. 4000 m, the terms of odorant binding and olfactory receptor activity were mainly enriched by up-regulated genes of *B. kashmirensis* but down-regulated by genes of *B. waltoni*.

### 3.3. KEGG Enrichment of DEGs

In the analysis of *B. kashmirensis*, 23,796 DEGs in samples from 4500 m were detected, among which 19,588 genes were up-regulated and 4208 genes were down-regulated compared to 4000 m. There were also 23,796 DEGs in samples from 5000 m, among which 563 genes were up-regulated and 23,233 genes were down-regulated compared to 4500 m. There were 10,379 DEGs in samples from 5000 m identified, among which 1669 genes were up-regulated and 8710 genes were down-regulated, compared to 4000 m. As for *B. waltoni*, there were 3721 DEGs in samples from 4500 m, with 2967 genes up-regulated and 754 genes down-regulated, compared to 4000 m. A total of 13,625 DEGs, including 4455 up-regulated genes and 9170 down-regulated genes, were detected in samples from 5000 m compared to 4500 m. There were 14,832 DEGs, including 10,859 up-regulated genes and 3973 down-regulated genes, recognized in samples from 5000 m compared to 4000 m.

**Figure 2:**
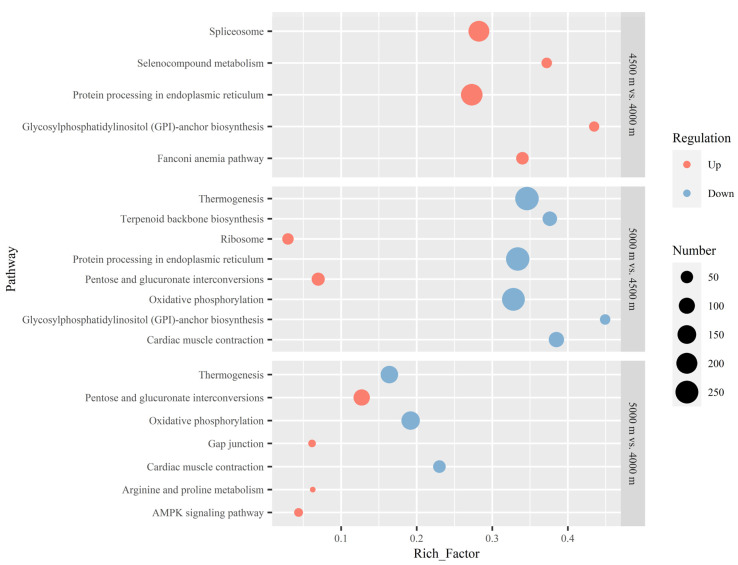
Pathways enriched by differentially expressed genes (DEGs) of *Bombus kashmirensis* in the comparisons between different altitudes. All pathways with an adjusted *p*-value lower than 0.05 are shown for each comparison. No significant down-regulated DEGs were detected in the 4500 m vs. 4000 m comparison; therefore, it is not included in the figure. “Number” denotes the number of DEGs enriched in the pathway. The pathways with red points indicate pathways enriched by up-regulated DEGs, while pathways with blue points are enriched by down-regulated DEGs.

In the pathway analysis of *B. kashmirensis*, for 4500 m vs. 4000 m, the Fanconi anemia pathway, a pathway related to DNA repair was enriched by up-regulated DEGs. There were no pathways enriched by down-regulated DEGs in this altitude comparison. As the altitude increased from 4500 m to 5000 m, the pentose and glucuronate interconversions pathway, which promotes carbohydrate metabolism, was enriched by up-regulated DEGs. Furthermore, the oxidative phosphorylation pathway—an aerobic glycolysis process—was enriched by down-regulated DEGs. For the 5000 m vs. 4000 m comparison, the pentose and glucuronate interconversions, gap junction, arginine and proline metabolism, and AMP-activated protein kinase (AMPK) signaling pathways were enriched by up-regulated DEGs. These pathways play roles in carbohydrate metabolism, neuroprotection in hypoxic tissues [33,34], amino acids metabolism, and cellular energy homeostasis, respectively. The oxidative phosphorylation pathway was enriched by down-regulated DEGs, as was the situation for 5000 m vs. 4500 m. As shown by the results, when the altitude increased from 4000 m to 4500 m, the DNA repair process was enhanced. When the altitude continued to increase to 5000 m, carbohydrate metabolism was enhanced while the aerobic respiration was weakened. When compared to *B. kashmirensis* from 4000 m, in the transcriptomes of bumblebees from 5000 m, the amino acids metabolism, carbohydrate metabolism, and cellular energy homeostasis processes were all promoted, while aerobic respiration was weakened (Figure 2).

**Figure 3:**
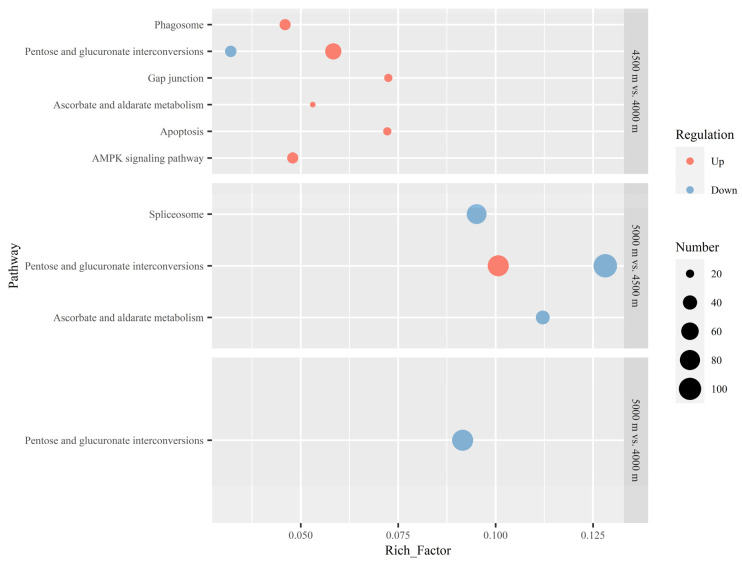
Pathways enriched by differentially expressed genes (DEGs) of *Bombus waltoni* in comparisons between different altitudes. All pathways with an adjusted *p*-value lower than 0.05 are shown for each comparison. No significant up-regulated DEGs were detected in the 5000 m vs. 4000 m comparison; therefore, it is not included in the figure. “Number” denotes the number of DEGs enriched in the pathway. The pathways with red points are enriched by up-regulated DEGs, while those with blue points are enriched by down-regulated DEGs.

In the pathway analysis of *B. waltoni*, for 4500 m vs. 4000 m, six pathways—the pentose and glucuronate interconversions, apoptosis, gap junction, AMPK signaling, phagosome, and ascorbate and aldarate metabolism pathways—were enriched by up-regulated DEGs. The pentose and glucuronate interconversions pathway was also enriched by down-regulated DEGs. A similar phenomenon was observed in the analysis of 5000 m vs. 4500 m. The pentose and glucuronate interconversions pathway was also enriched by both up- and down-regulated DEGs, all with more DEGs and to a greater degree than the 4500 m vs. 4000 m comparison. Another two pathways—the spliceosome and the ascorbate and aldarate metabolism pathways—were also detected to be enriched by down-regulated DEGs in this altitude shift. In the 5000 m vs. 4000 m comparison, the pentose and glucuronate interconversions pathway was again enriched by downregulated DEGs, while there were no other pathways enriched by up- or down-regulated DEGs in this comparison. There were some pathways that were up-regulated with increasing elevation in the results of both species, but the up-regulation of these pathways occurred in different altitude-change situations for the two species. For *B. kashmirensis*, the pentose and glucuronate interconversions, gap junction, and AMPK signaling pathways were up-regulated at the high altitude of 5000 m, while they were already detected to be enhanced at 4500 m for *B. waltoni* (Figure 3).

## 4. Discussion

Determining the mechanisms by which bumblebees adapt to high altitudes can help us to better understand their distribution, allowing for further prediction of future range shifts driven by climate change, which may serve as a basis for the protection and utilization of bumblebee resources. In this study, the northeastern QTP was selected as the study area, and two dominant and widespread bumblebee species—*B. kashmirensis* and *B. waltoni*—were selected as the study subjects. Differing from previous bumblebee transcriptome studies, here, the distances and altitude differences between collection sites were respectively controlled to no more than 100 km and 1000 m, in order to minimize the influence of genetic differences possibly caused by great geographical distances or altitude differences on the transcriptomic results.

As shown in our results, bumblebees tended to up-regulate energy metabolism-related genes in extremely high-altitude environments. Many pathways closely related to energy production were significantly up-regulated in both studied bumblebee species with increasing altitude. The pentose and glucuronate interconversions pathway, which is an important component of carbohydrate metabolism, was enriched by up-regulated DEGs in *B. kashmirensis* at the high altitude of 5000 m, compared to 4000 m and 4500 m (Figure 2). The AMPK signaling pathway, which has positive effects on cellular energy homeostasis by maintaining sufficient adenosine triphosphate (ATP) resources, was observed to be enhanced in both species with increasing altitude: from 4000 m to 5000 m for *B. kashmirensis* (Figure 2) and from 4000 m to 4500 m for *B. waltoni* (Figure 3). Additionally, enrichment by up-regulated DEGs from the arginine and proline metabolism pathways—both of which belong to the amino acids metabolism category—were detected in *B. kashmirensis* in the 4500 m vs. 4000 m (Figure 2) and 5000 m vs. 4000 m (Figure 2) comparisons, respectively. As implied by the above results, the energy production of bumblebees was obviously strengthened with increasing altitude. The reason why energy production should be enhanced may be related to the increasing needs for heat and flight power with increasing altitude. From the perspective of heat, as the ambient temperature will consistently decrease with the rising elevation, increased convective heat loss is inevitable when bumblebees live in colder environments at higher altitudes. Thus, the bumblebees must produce more heat through metabolism and utilization of ATP [27,30,35]. Enhancement of energy substance metabolism and the AMPK signaling pathway can meet the increasing heat demand of bumblebees with increasing altitude. From the perspective of flight power, the forces produced by insects when flapping their wings are directly proportional to air density [22]. The low air density at higher altitudes is a great disadvantage of flight [36,37]. At higher altitudes, insects have to use more energy to flap their wings more frequently, in order to the aerodynamic forces required to offset their body weight [38,39]. Thus, bumblebees up-regulate energy metabolism-related genes as the altitude increases.

In addition to the low temperature and low air density mentioned above, bumblebees at high altitudes are still faced with another great challenge: hypoxia. Liu et al. [25] have considered high-altitude adaptation under a tremendous altitude difference of over 4000 m, and reported that the high-altitude bumblebees had to enhance their aerobic respiration (e.g., through the oxidative phosphorylation pathway), in order to provide enough energy to ensure normal flight, compared to the low-altitude bumblebees. In this study, we seemed to observe a completely different phenomenon. Compared to 4000 m and 4500 m, oxidative phosphorylation was enriched by down-regulated DEGs in *B. kashmirensis* at the high altitude of 5000 m (Figure 2). The paradox regarding the adaptive change of oxidative phosphorylation to the hypoxia may be attributed to the difference in the level of altitude shift considered in the two studies and the limitation of the adaptation of respiration processes and oxidative phosphorylation. In responding to hypoxia, to some extent, insects can alter their respiratory system [36]. They can increase the number of terminal tracheal branches [40,41] and the diameter of primary tracheae, in order to compensate for the hypoxic environment [42,43,44]; however, the reasonable allocation of muscle fibers, mitochondria, tracheae, and sarcoplasmic reticulum in the thorax volume limits such an increased respiratory system [45]. Thus, to a certain degree, insects can adapt to the hypoxic environment by strengthening their respiratory system and oxidative phosphorylation, in line with the result of Liu et al. [25]. However, when the increased respiratory system still fails to provide enough oxygen under extreme hypoxia at extremely high altitudes, the adaptation mechanism may change and the aerobic respiration is abated to avoid redundancy as much as possible. Here, as our study was aimed at determining the adaptation to extremely high altitudes in the alpine region in the northeastern QTP, all three sampling sites were designed at altitudes no less than 4000 m, which may be close to the limit of respiratory system and oxidative phosphorylation adaptation [46]. Oxidative phosphorylation was enriched by down-regulated DEGs at the high altitude of 5000 m.

As indicated by the above differences in the results of the different comparisons, there were some gradual changes as the altitude increased. For example, in the analysis of *B. kashmirensis*, the up-regulation of arginine and proline metabolism and the up-regulation of the AMPK signaling pathways were not significant in the comparisons of 4500 m vs. 4000 m and 5000 m vs. 4500 m but were significant in the comparison of 5000 m vs. 4000 m (Figure 2). This implied the gradual changes in the two processes when the elevation changes from 4000 m to 5000 m of the two processes. Meanwhile, some processes demonstrated transcriptomic changes to quite different degrees under different situations of increased altitude. This was also well indicated by the different comparisons considered in this study; for example, in the analysis of *B. kashmirensis*, the pentose and glucuronate interconversions pathway was greatly up-regulated in the comparison of 5000 m vs. 4500 m but not in the comparison of 4500 m vs. 4000 m (Figure 2). The down-regulation of oxidative phosphorylation demonstrated a similar trend (Figure 2). This suggests that there are more greater changes in these processes in *B. kashmirensis* for them to respond to the more extreme altitude increasing. In the analysis of *B. waltoni*, the AMPK signaling pathway was up-regulated in the comparison 4500 m vs. 4000 m but not regulated in comparisons 5000 m vs. 4500 m and 5000 m vs. 4000 m (Figure 3). This might be relative to the adaptation limit of *B. waltoni*. Although *B. waltoni* could survive at 5000 m, this extreme high-altitude environment might pose a great challenge and even exceed its adaptation limit. Energy regulations could not meet its original energy-intensive activity. Then, *B. waltoni* has to limit its own activity, with the AMPK signaling pathway being down-regulated indistinctively in the comparison of 5000 m vs. 4500 m.

It can also be inferred that the mechanisms of extremely high-altitude adaptation for the two bumblebee species (i.e., *B. kashmirensis* and *B. waltoni*) differed. Compared to cospecies at 4000 m, *B. kashmirensis* at 5000 m showed up-regulated pentose and glucuronate interconversions pathway, arginine and proline metabolism pathway, and AMPK signaling pathway (Figure 2) while *B. waltoni* at 5000 m showed a down-regulated pentose and glucuronate interconversions pathway and no up-regulated energy metabolism (Figure 3). This may be due to the difference between the two species, regarding their need for energy in response to altitude changes. Aside from the difference regarding the regulation of energy metabolism, there were also differences between the adaptation mechanisms of these species on other aspects. Peroxidation stress can be seen as a common challenge caused by the hypoxia at high altitudes, and DNA repair and apoptosis have been interpreted as two physiological processes related to the response to peroxidation stress [47]. When the altitude increased from 4000 m to 4500 m, *B. kashmirensis* enhanced its DNA repair function (Figure 2) while *B. waltoni* strengthened the apoptosis process (Figure 3). Furthermore, for *B. kashmirensis*, the gap junction and AMPK signaling pathways were up-regulated at the high altitude of 5000 m (Figure 2) while they were already detected to be enhanced at 4500 m in *B. waltoni* (Figure 3). These results may provide a clue about the different adaptations of two bumblebee species to extremely high altitudes. The different adaptation might be related to genetic background. Bumblebees belonging to different subgenera had different genetic strategies for adapting to high altitudes [23]. The different genetic strategies made them have different transcriptional regulation processes to respond to high altitudes [25]. The genetic differences between the two species considered here resulted in the inherent differences in their transcriptional regulation. The extreme high altitude further resulted in their transcriptional regulation being more targeted in order to meet their own needs more effectively. Therefore, there were many differences regarding the adaptation demonstrated via transcriptional regulation between the two species. However, to obtain a definite explanation regarding these phenomena, larger sample sizes and more comprehensive sampling covering the whole altitude range of the two species, as well as investigations of their distribution patterns, are necessary.

## 5. Conclusions

We showed, according to the results of our study, that the considered bumblebees tend to up-regulate energy metabolism-related genes when adapting to extremely high-altitude environments. Of the enriched pathways up-regulated in higher altitudes, the pentose and glucuronate interconversions pathway presented the most severe up-regulation in multiple comparisons at different altitudes for *B. kashmirensis*, while the AMPK signaling pathway was up-regulated in both species. However, due to the extreme hypoxia in this study, oxidative phosphorylation was detected to be down-regulated as the altitude kept increasing, which is uncommon in studies on bumblebee adaptation to high altitudes.

## Figures and Tables

**Table 1:** Data quality and mapping ratios for all samples.

Sample	Raw Reads	Clean Reads	Clean Bases (G)	Q30 (%)	Mapping Ratio (%)
KH1	49,297,998	48,807,124	6.68	93.53	76.12
KH2	46,165,938	45,665,128	6.27	93.57	69.74
KH3	51,256,632	50,814,744	6.94	93.85	73.49
KH4	52,799,682	52,316,554	7.12	93.75	76.73
KH5	51,856,992	51,368,296	7.04	93.47	74.33
KH6	54,244,072	53,759,034	7.37	93.50	75.81
KH7	44,083,406	43,501,182	5.96	92.98	71.97
KM1	48,574,042	48,087,392	6.63	93.46	77.37
KM2	44,543,734	44,187,246	6.11	93.77	77.92
KM3	45,822,524	45,421,310	6.28	93.66	78.59
KM4	43,216,750	42,745,790	5.92	93.65	74.83
KM5	51,397,656	50,997,892	7.07	93.79	76.58
KL1	46,079,538	45,670,082	6.32	94.07	78.60
KL2	44,536,062	44,243,768	6.12	93.69	78.77
KL3	48,138,454	47,609,454	6.53	93.74	76.33
KL4	53,660,950	53,159,194	7.27	94.01	83.32
KL5	41,377,256	40,918,026	5.62	93.34	74.40
WH1	45,484,192	45,036,622	6.21	93.74	74.02
WH2	43,073,130	42,713,976	5.92	92.14	76.93
WH3	43,477,462	42,996,858	5.95	93.50	76.23
WH4	48,568,010	48,144,898	6.66	93.68	74.11
WH5	52,250,474	51,806,312	7.17	93.71	75.00
WM1	52,761,382	52,233,550	7.23	93.45	75.00
WM2	50,270,968	49,854,368	6.88	93.97	75.44
WM3	51,491,370	51,017,398	7.07	93.78	74.25
WL1	49,706,148	49,026,188	6.72	93.79	77.19
WL2	45,457,166	44,781,906	6.16	93.84	82.28
WL3	63,441,978	62,100,090	7.74	93.45	74.63
WL4	52,275,552	51,754,244	7.13	93.87	75.41
WL5	49,883,828	49,414,792	6.81	94.07	74.79

In the sample labels, K and W represent samples of *B. kashmirensis* and *B. waltoni*, respectively. The second letters H, M, and L in the sample labels indicate the 5000 m, 4500 m, and 4000 m sampling altitudes respectively. Q30 denotes the number of clean reads.

**Table 2:** Results of the gene set enrichment analysis for *Bombus kashmirensis*.

4500 m vs. 4000 m		5000 m vs. 4500 m		5000 m vs. 4000 m	
Enriched GO term	NES	Enriched GO term	NES	Enriched GO term	NES
RNA-directed DNA polymerase activity	−2.78	**G-protein coupled receptor activity**	3.00	**Odorant binding**	2.18
Ligand-gated channel activity	−2.47	Structural constituent of cuticle	2.90	**Olfactory receptor activity**	2.16
Ligand-gated ion channel activity	−2.25	Transmembrane signaling receptor activity	2.89	**G-protein coupled receptor activity**	2.13
Extracellular ligand-gated ion channel activity	−2.25	Signaling receptor activity	2.89	Transmembrane signaling receptor activity	2.12
Motile cilium	−2.03	Ligand-gated ion channel activity	2.86	Signaling receptor activity	2.18
Glutamate receptor activity	−2.00	Ligand-gated channel activity	2.82	Intra-Golgi vesicle-mediated transport	−2.11
Neurotransmitter receptor activity	−1.95	Extracellular ligand-gated ion channel activity	2.70	Protein kinase complex	−2.09
**Odorant binding**	−1.94	Extracellular region	2.65	Positive regulation of nucleic acid-templated transcription	−2.07
S-methyltransferase activity	1.92	**Odorant binding**	2.58	Positive regulation of RNA metabolic process	−2.07
Ion channel complex	−1.91	Molecular transducer activity	2.53	Cyclin-dependent protein kinase holoenzyme complex	−2.07
Structural constituent of cuticle	−1.90	Neurotransmitter receptor activity	2.49	Positive regulation of transcription, DNA-templated	−2.05
Protein lipidation	1.90	**Olfactory receptor activity**	2.47	Positive regulation of RNA biosynthetic process	−2.05
**G-protein coupled receptor activity**	−1.89	Receptor ligand activity	2.40	Endoplasmic reticulum-Golgi intermediate compartment	−2.02
5-methyltetrahydropteroyltri-L-Glutamate-dependent methyltransferase activity	1.89	Hormone activity	2.40	Positive regulation of transcription by RNA polymerase II	−2.01
Ionotropic glutamate receptor activity	−1.88	Signaling receptor binding	2.39	Positive regulation of nucleobase-containing compound metabolic process	−2.00
Chitin binding	−1.88	Cell–cell adhesion	2.35	Meiosis I cell cycle process	−1.99
**Olfactory receptor activity**	−1.87	Transmitter-gated ion channel activity	2.35	Positive regulation of gene expression	−1.98
Transmitter-gated channel activity	−1.87	Homophilic cell adhesion via plasma membrane adhesion molecules	2.34	COPI vesicle coat	−1.98
Steroid dehydrogenase activity	1.87	Motile cilium	2.34	Acylglycerol O-acyltransferase activity	1.97
5-methyltetrahydropteroyltriglutamate-homocysteine S-methyltransferase activity	1.86	Transmitter-gated channel activity	2.33	Regulation of DNA-templated transcription, elongation	−1.97

The samples from the lower altitude were set as the control group in each analysis. The enriched gene ontology (GO) terms with the top 20 largest absolute normalized enrichment score (NES) are shown here. A positive NES indicates that the genes associated to this term were mainly upregulated in the higher altitude while a negative NES indicates the opposite. The terms shared in the three comparisons are in bold. The genes that contributed significantly to these terms are in Table S1.

**Table 3:** Results of the gene set enrichment analysis for *Bombus waltoni*.

4500 m vs. 4000 m		5000 m vs. 4000 m	
GO term	NES	GO term	NES
Extracellular region	−2.64	Regulation of chemotaxis	−3.12
Motile cilium	−2.61	Regulation of locomotion	−2.55
Structural constituent of cuticle	−2.50	Response to herbicide	−2.48
**Odorant binding**	−2.45	snoRNA binding	−2.36
Cilium	−2.42	rRNA modification	−2.30
1-acyl-2-lysophosphatidylserine acylhydrolase activity	−2.32	Electron transporter, transferring electrons within the cyclic electron transport pathway of photosynthesis activity	−2.37
Fatty-acyl-CoA reductase (alcohol-forming) activity	−2.29	**Odorant binding**	−2.11
Alcohol-forming fatty acyl-CoA reductase activity	−2.28	Regulation of response to external stimulus	−2.07
Phospholipase A1 activity	−2.28	Cytosolic ribosome	−1.96
Phosphatidylserine 1-acylhydrolase activity	−2.26	Demethylation	2.08
Chitin binding	−2.25	Olfactory receptor activity	−1.90
Signaling receptor activity	−2.25		
Cell junction	−2.23		
Cell projection	−2.23		
Dynein complex	−2.22		
Transmembrane signaling receptor activity	−2.18		
Triglyceride lipase activity	−2.16		
Plasma membrane bounded cell projection	−2.15		
Cyclic-nucleotide phosphodiesterase activity	−2.06		
Wnt signaling pathway	−2.06		

The samples from the lower altitude were set as the control group in each analysis. The enriched Gene ontology (GO) terms with the top 20 largest absolute normalized enrichment score (NES) are shown here. Columns for 5000 m vs. 4500 m are absent, as no significant GO terms were found in this comparison. The meanings of the NES values are same as in Table 2. The terms shared in the two comparisons are in bold. The genes that contributed significantly to these terms are in Table S1.

## Data Availability

The transcriptomic data have been deposited to NCBI SRA database. And the accession number as follows: *Bombus kashmirensis*: from SRR19913284 to SRR19913300, *Bombus waltoni*: from SRR19913147 to SRR19913159. The expression values of bumblebees’ transcript have been deposited to figshare URL: DOI of *Bombus kashmirensis*: 10.6084/m9.figshare.21202127, DOI of *Bombus waltoni*: 10.6084/m9.figshare.21202145.

## Supplementary Materials

The following supporting information can be downloaded at: https://www.mdpi.com/article/10.3390/insects13121173/s1. Table S1: The genes that contributed significantly to the enrichment score in the gene set enrichment analysis.
